# A Case of Idiopathic Tetanus

**Published:** 1847-02

**Authors:** W. Wadsworth

**Affiliations:** Racine, Wis. Ter.


### ARTICLE II.

*A Case of Idiopathic Tetanus.* By W. Wadsworth, M.D., of Racine, Wis. Ter.

M. A. Palmer, Esq., of sanguine nervous temperament, aged 26, a lawyer by profession, had his constitution considerably impaired by successive fevers, which he suffered some years since, at the South. He, howevever, so far recovered as to be able to pursue his studies, and engaged in the practical duties of his profession.

I was called to see him on the 18th of June last, and found him suffering under a recent attack of bilious cholic, with the usual symptoms—obstinate constipation of the bowels, severe pain, nausea, and occasional vomiting; the pulse but little disturbed. Opium in full doses, combined with calomel and repeated; with warm fomentations to the abdomen, subdued the pain, and the constipation was finally overcome by castor and croton oil; and in a few days he was able to resume the business of his profession. From this time up to his recent illness, he occasionally complained of more or less irregularity of bowels, capricious appetite, and sometimes of great exhaustion from a little over-exertion. For these difficulties he took, occasionally, the blue pill, mild tonics, and port wine.

On the evening of the 1st of September I was called again to see him. Found he had slight chill, severe pain and soreness of head, coated tongue, entire loss of appetite—in fact, all the incipient symptoms of an attack of bilious remittent fever, which was prevailing extensively around him at the time. Gave an emetic of ipecac., to be followed by powders of James and Dover, with 4 grs. calomel every four hours, and the acetate ammonia every four hours. The next morning he had less pain of head, some uneasiness about the neck of the bladder, and difficulty of making water. A cathartic of senna and rhœi was given, with mucilaginous drinks, and sweet spirits of nitre every two hours.

In the after part of the day found him with considerable nervous irritation. The cathartic had not operated, and, the difficulty of making water continuing with augmented force, ordered a stimulating enema, the introduction of which gave him great pain at the time. It was, however, soon followed by free evacuation, indicative of a bad state of the secretions. Warm fomentations were applied to the region of the bladder, and uva ursi, juniper, and gum arabic, was given, to which, in a little time, the buchu was added, with marked success over the urinary difficulty, and he experienced no further trouble in this respect during the whole course of the disease.

After this several days passed with but a mild grade of fever: the secretions were endeavoured to be improved with small doses of calomel, and the hydrarg. cum creta, with mild cathartics, which, by the by, always produced *more than or-*

*dinary irritation in their operation*, but which was easily allayed by Dover or morphine that was used for this purpose. Under this course the fever soon declined, and convalescence seemed to be fairly advancing. His spirits, which during the first part of his sickness were at times greatly depressed, were now more buoyant, the tongue becoming clean, the appetite improving, and every thing indicating that, at least, his usual health would soon be established.

But this bright prospect was soon obscured and darkened by a train of symptoms as *unique* as they were inexplicable. At noon on the 10th, while taking his dinner, he complained of some stiffness and inability to move his jaws. He was directed to wash them in some warm spirits of camphor and laudanum, and encouraged to hope that it would soon disappear. In the evening the symptoms still continuing, particular enquiries were instituted whether any local irritation existed about the mouth, *teeth, or any other part, and finding none, and the general system undisturbed,* I could not bring myself to the belief that it was any thing of the nature of that terrible disease which it soon, notwithstanding, proved itself to be. The treatment of quinine and wine was continued, and a full dose of morphia and camphor at bed time. The jaws were bathed in a strong stimulating liniment through the night. He slept well during the night, and up to this time the pulse was undisturbed, beating but eighty to the minute, full and soft, no fever, nor the slightest pain or irritation in any part of the system—save, indeed, some anxiety of mind as to the singularity and obstinacy of the difficulty about his jaws.

Soon after this, however, the difficulty of deglutition, and especially in the swallowing of liquids, and rigidity and drawing backward of the muscles of the neck, with slight general spasms, announced, in a manner not to be misunderstood, the terrible nature of the disease. The pulse was now increased in frequency, the tongue was more coated, and the discharge from the bowels was liquid, dark coloured and extremely fetid. Blisters were applied to the jaws and side of the neck; morphia, 1 gr. with 5 of calomel and morphia in grain doses, with camphor, directed every two hours; and wine, in free and large doses, given through the night. But these means produced not the slightest control over the spasms.

The night was passed without sleep or rest, the patient often remarking that "if those spasms could be stopped he should be perfectly well." He also complained of being hungry. In the mean time the abdominal muscles became retracted and perfectly hard; the pulse 120 and weaker; the power of deglutition more embarrassed, and sometimes almost lost; the surface covered with an abundant perspiration; the spasms more frequent and more intense. Dr. Graves and several other medical friends were called in in consultation. A more decided tonic and narcotic treatment was recommended. 15 grains of quinine, 4 of opium, and 6 of capsicum was given; the quinine to be repeated in two hours, and tea spoonful doses of laudanum to be given every hour; tincture of nux vomica 20 gtt. every two hours. To these was added, after a little time, the prussic acid, 4 gtt. every two hours.

These powerful and often repeated medicines had not the slightest control over the spasms, nor did they in the least manifest their narcotic effects upon the brain or nervous system—the mind remaining clear and unclouded to the last. As the symptoms advanced the breathing became more and more embarassed; a livid palor overspread the countenance; a clammy sweat, the surface; the pulse rapidly sinking, while the spasms were increasing in frequency and power, till a general convulsion of the whole muscular system terminated at once the sufferings and life of the patient in 48 hours from the first notice of any difficulty about the jaws.

Idiopathic Tetanus is a disease rarely seen at the north, and the causes that gave rise to it in this case are very obscure. It is a matter of regret that a post mortem examination was not obtained. It might have revealed some internal lesion, which though not announced by any marked symptom, was sufficient, in a *constitution peculiarly excitable* as was his, to have brought it on. The disease of the bowels he had recently suffered, as well as the inordinate irritation produced by the mildest purgatives during his illness, would seem to indicate that intestinal irritation, or some structional lesion in some portion of the alimentary tube, was more than probably, the source of this terrible malady.

